# Digital endpoints as outcome measures in orthopaedic care and their psychometric properties: a scoping review protocol

**DOI:** 10.1136/bmjopen-2026-117633

**Published:** 2026-11-03

**Authors:** Reem Fakak, Sultan Aslam, Benjamin Davies, Saif Hadi, Ali Gharooni

**Affiliations:** 1 University of Nicosia Medical School, Nicosia, Cyprus; 2 School of Clinical Medicine, University of Cambridge, Cambridge, UK; 3 Academic Neurosurgery Unit, Department of Clinical Neurosciences, University of Cambridge, Cambridge, UK; 4 Department of Trauma and Orthopaedics, Royal Blackburn Hospital, Blackburn, UK

**Keywords:** Wearable Electronic Devices, Orthopaedic & trauma surgery, REHABILITATION MEDICINE, Psychometrics, ORTHOPAEDIC & TRAUMA SURGERY, Orthopedics

## Abstract

**Abstract:**

**Introduction:**

Digital health technologies, including wearable sensors, smartphone-based assessments and vision-based systems, are increasingly used to generate objective outcome measures in orthopaedic care. These digital endpoints have the potential to complement traditional patient-reported outcome measures by providing continuous, objective assessments of functional recovery in real-world settings. However, the extent to which these endpoints have undergone psychometric validation remains unclear, and existing reviews are limited to specific conditions or technologies. This scoping review aims to systematically map the range of digital endpoints used in orthopaedic care and synthesise the available evidence regarding their psychometric properties

**Methods and analysis:**

This scoping review will be conducted in accordance with the Joanna Briggs Institute methodology for scoping reviews and reported following the Preferred Reporting Items for Systematic Reviews and Meta-Analyses extension for Scoping Reviews (PRISMA-ScR). MEDLINE (Ovid), Embase, CINAHL, IEEE Xplore, Web of Science and the Cochrane Library will be searched for studies published from January 2005 to December 2025. Searches are expected to be completed in February 2026. Eligible studies will include paediatric and adult populations with orthopaedic conditions evaluating at least one psychometric property of a digital endpoint, including validity, reliability, responsiveness or interpretability. Two reviews will independently perform study selection and data extraction using a Consensus-based Standards for the selection of health Measurement Instruments (COSMIN)-informed extraction tool, with disagreements resolved through discussion or a third reviewer. Extracted data will be synthesised descriptively and presented using tables, figures and narrative summaries to map the current evidence base and identify research gaps. The review is planned to commence in February 2026 and conclude in September 2026.

**Ethics and dissemination:**

Ethics approval is not required because this review will synthesise data from published literature without involving human participants. Findings will be disseminated through publication in a peer-reviewed journal and presentation at relevant scientific meetings.

**Registration:**

Open Science Framework: https://osf.io/n4ctu

**STRENGTHS AND LIMITATIONS OF THIS STUDY**

- Uses Joanna Briggs Institute methodology and Preferred Reporting Items for Systematic Reviews and Meta-Analyses extension for Scoping Reviews (PRISMA-ScR) guidance to ensure transparent and reproducible review methods.
- Searches multiple medical, rehabilitation and engineering databases to maximise identification of relevant studies.
- Applies a Consensus-based Standards for the selection of health Measurement Instruments (COSMIN)-informed framework to classify psychometric properties consistently across heterogeneous digital endpoints.
- Independent duplicate screening and data extraction will minimise selection and extraction bias.
- Restriction to English-language publications may exclude relevant evidence published in other languages.

## Introduction

 Outcome measurement in orthopaedics has traditionally relied on patient-reported outcome measures (PROMs) to determine the efficacy of an intervention.^1^ While PROMs provide valuable insight, the impact of an illness on a person, they are subject to notable limitations, such as recall bias, ceiling effects, measurement variability and regression to the mean.^2–4^ They can be augmented by secondary objective outcomes such as range of motion, radiographic parameters and activity levels; however, these will often require face-to-face assessment which increases trial costs and footprint. These limitations may limit accurate tracking of recovery, impair cross-study comparability, inflate measurement error in high-functioning or rapidly recovering populations and increase trial costs and feasibility.^5–7^

Digital endpoints, derived from wearable sensors, smartphone applications, inertial measurement units (IMUs), camera-based systems and sensor-enabled rehabilitation platforms, have emerged as an innovative, objective means to monitor real-world functional outcomes.^8–11^ They may be able to quantify gait, mobility, range of motion, activity levels, balance and motor behaviour with high granularity and ecological validity.^12^

A scoping review was chosen because the objective is to comprehensively map the range of digital endpoints and their psychometric evaluation across orthopaedic care rather than to answer a narrowly defined effectiveness question or synthesise intervention effects. This approach is recommended by the Joanna Briggs Institute (JBI) methodology when evidence is heterogeneous and aims include identifying research gaps and informing future systematic reviews.

This scoping review will attempt to address this gap and inform ongoing work on digital biomarkers and outcome measurement frameworks, including the team’s efforts to expand digital outcome into the broad orthopaedic field.

### Review question

Which digital endpoints are currently used as outcome measures in orthopaedic care, and what is the extent and nature of their psychometric validation (validity, reliability, responsiveness and interpretability)?

### Inclusion criteria

#### Participants

The proposed scoping review will include studies involving individuals of any age with orthopaedic conditions, including both adult and paediatric populations.

The focus will be to map and classify digital endpoints used as outcome measures in orthopaedic care and summarise their psychometric properties (validity, reliability, responsiveness, interpretability). Eligible participants will include those undergoing surgery for fracture fixation, ligament reconstruction, spine procedures, arthroplasties or upper and lower limb surgeries. Individuals managed non-operatively for conditions including degenerative joint disease, sports injuries, trauma and congenital or developmental orthopaedic conditions will also be included. The broad inclusion of orthopaedic populations reflects the increasing application of digital endpoints across the full spectrum of clinical presentations. Studies will be excluded if the focus is solely on non-orthopaedic conditions (eg, rheumatological diseases like rheumatoid arthritis, or neurological conditions like stroke), as the functional movement patterns in these populations differ significantly from orthopaedic patient.

#### Concept

This review will consider studies evaluating digital endpoints as outcome measures. For the purpose of this review, digital endpoints are defined as objective, quantifiable physiological or functional measures derived from digital technologies.^13 14^ This includes wearable IMUs, accelerometers, smartphone sensors, vision-based motion analysis systems or smart implants, which assess mobility, functional performance, range of motion or other movement-related behaviours relevant to orthopaedic care. Studies must assess at least one psychometric property to demonstrate suitability for clinical research or practice. In alignment with the Consensus-based Standards for the selection of health Measurement Instruments (COSMIN) framework, these properties specifically include: reliability (including inter-rater/intra-rater reliability and measurement error), validity (including criterion, construct and concurrent validity), responsiveness to clinical change and interpretability (such as the Minimal Clinically Important Difference or Minimal Detectable Change). Studies which employ digital tools purely for descriptive observation without psychometric assessment or those involving platforms solely measuring patient-reported information without objective sensor-derived metrics, will be excluded to maintain adherence with the review objective.

#### Context

The proposed review will consider validation studies, measurement property studies and cohort studies that report a psychometric subanalysis. Studies conducted in clinical, rehabilitation, home-based or community settings where digital endpoints are applied to assess function in real-world or clinically relevant environments, will also be considered. This means that while wearable sensors used in home environments or outpatient clinics will be included, studies relying exclusively on complex, stationary laboratory equipment (such as marker-based 3D optical motion capture systems fixed within a biomechanics lab) will be excluded, as these cannot be deployed as routine digital endpoints in standard orthopaedic clinical trials or remote patient monitoring. There will be no geographic restrictions. Studies published in languages other than English will be excluded due to resource limitations.

#### Types of sources

This scoping review will include analytical and validation-focused study designs that evaluate the psychometric properties of digital endpoints in orthopaedic care. Eligible study designs may include randomised controlled trials, non-randomised controlled studies, prospective and retrospective cohort studies, case-control studies and analytical cross-sectional studies. This is provided that they report at least one psychometric property such as validity, reliability, responsiveness or interpretability.

Studies that are purely descriptive in nature, including feasibility-only studies and qualitative research without quantitative evaluation of psychometric properties, will be excluded. Case reports, case series and opinion papers that do not include psychometric analysis will also be excluded.

Systematic reviews will not be included as primary evidence sources, but their reference lists may be screened to ensure identification of relevant primary research. This ensures the review focuses on validated digital metrics.

The inclusion and exclusion criteria used for study selection are summarised in table 1.

**Table 1:** Inclusion and exclusion criteria used for screening articles

Domain	Include	Exclude
Population	Individuals of any age with orthopaedic conditions, surgical or non-surgical	Non-orthopaedic conditions or studies without clear relevance to orthopaedics
Concept	Digital endpoints providing objective, sensor-derived measures of functional performance or movement. Must evaluate one or more psychometric properties (validity, reliability, responsiveness, interpretability)	Digital tools used only descriptively without psychometric analysis and providing only patient-reported measures without objective data
Context	Clinical, rehabilitation, home-based or community settings with real-world or clinically applicable measurement	Laboratory-based instrumented gait assessments without clinical relevance
Study design	Analytical and validation-oriented designs (eg, RCTs, non-randomised studies, cohort studies, case-control studies, analytical cross-sectional studies)	Case reports, case-series, feasibility-only studies, qualitative-only evidence, expert opinion, narrative reviews
Evidence source	Primary research studies evaluating psychometric properties. Reference lists of systematic reviews may be used for additional records	Systematic reviews and meta-analyses as primary evidence
Language	English only	Non-English studies
Publication date	2005 or later	Pre-2005 studies

## Methods

This proposed scoping review will be conducted in accordance with the JBI Scoping Review Methodology and reported according to PRISMA-ScR.^15 16^ The protocol has been registered on the Open Science Framework (OSF). The study is planned to commence in February 2026 and conclude in September 2026. Database searches will include studies published up to December 2025 and are expected to be completed in February 2026.

### Search strategy

A three-step search strategy will be used, consistent with the JBI guidance. An initial limited search of MEDLINE (Ovid) is to be conducted to identify relevant studies and refine search terminology based on text words in titles or abstracts, and index terms used to describe the studies. A full search strategy will then be synthesised using relevant keywords and subject headings and adapted for each included database (online supplemental file 1).

Searches will be conducted in the following databases to ensure broad coverage of medical, rehabilitation and digital technology research: MEDLINE (Ovid), Embase, CINAHL (EBSCO), IEEE Xplore, Web of Science and Cochrane Library. Studies published from January 2005 to December 2025 will be included to reflect the modern era of digital health technology adoption in orthopaedics. Studies will be limited to English-language publications due to resource limitations. The reference lists of included studies and relevant reviews will be screened to identify any additional eligible studies not captured in the database search.

### Source selection

All identified records will be collated and uploaded into a citation management system (Covidence), and duplicates will be removed. Two independent reviewers will conduct title and abstract screening, followed by full-text screening of potentially relevant articles. Reasons for exclusion at full-text screening will be recorded and reported. Disagreements will be resolved by a third reviewer or discussion. The results of the search and selection process will be presented in the final review using a PRISMA-ScR flow diagram.

### Data extraction

Data will be extracted independently by two reviewers using a draft extraction tool developed for this review using Microsoft Excel. The tool will capture details related to study characteristics, digital endpoint device, measurement approach, psychometric properties and clinical applicability. Psychometric data will be extracted and coded according to the COSMIN framework.^17^ The draft tool will be modified and revised during data extraction, with any revisions documented. Authors of included studies may be contacted for clarification or to obtain missing psychometric information.

### Data analysis and presentation

Extracted data will be synthesised descriptively and mapped within the population-concept-context framework. Findings will be presented in tabular and graphical formats, such as summary matrices of digital endpoints by clinical domain and evidence maturity. A narrative synthesis will accompany tables and figures to describe how the results address the scoping objectives. No meta-analysis will be undertaken due to anticipated heterogeneity in digital measurement approaches and endpoints.

## Supplementary material

10.1136/bmjopen-2026-117633 online supplemental file 1

## References

[1] Cella MS, Baumhauer JF, Rothrock NE (2025). Use of Patient-Reported Outcomes Measurement Information System Measures in Orthopaedic Specialties: Results of a Scoping Review for 2018 to 2022. J Am Acad Orthop Surg.

[2] Braaksma C, Wolterbeek N, Veen MR (2024). Assessing the measurement properties of PROMIS Computer Adaptive Tests, short forms and legacy patient reported outcome measures in patients undergoing total hip arthroplasty. *J Patient Rep Outcomes*.

[3] MARS Group (2025). Relationships Between PROMIS and Legacy Patient-Reported Outcome Measure (PROM) Scores in the MARS Cohort at 10-Year Follow-up. J Bone Joint Surg Am.

[4] Wamper KE, Sierevelt IN, Poolman RW (2010). The Harris hip score: Do ceiling effects limit its usefulness in orthopedics?. Acta Orthop.

[5] Spece H, Kurtz MA, Piuzzi NS (2025). Patient-reported outcome measures offer little additional value two years after arthroplasty : a systematic review and meta-analysis. Bone Joint J.

[6] van Gerven P, van Dongen JM, Rubinstein SM (2020). Reduction of routine use of radiography in patients with ankle fractures leads to lower costs and has no impact on clinical outcome: an economic evaluation. BMC Health Serv Res.

[7] Birir A, Amen TB, Varady NH (2021). Clinical efficacy and cost-effectiveness of postoperative radiographs after total knee arthroplasty. Knee.

[8] Coravos A, Khozin S, Mandl KD (2019). Developing and adopting safe and effective digital biomarkers to improve patient outcomes. *NPJ Digit Med*.

[9] Kobsar D, Masood Z, Khan H (2020). Wearable Inertial Sensors for Gait Analysis in Adults with Osteoarthritis-A Scoping Review. Sensors (Basel).

[10] Taborri J, Palermo E, Rossi S (2016). Gait Partitioning Methods: A Systematic Review. Sensors (Basel).

[11] FDA-NIH Biomarker Working Group (2016). BEST (Biomarkers, EndpointS, and other Tools) resource.

[12] Rose MJ, Costello KE, Eigenbrot S (2022). Inertial Measurement Units and Application for Remote Health Care in Hip and Knee Osteoarthritis: Narrative Review. *JMIR Rehabil Assist Technol*.

[13] Tackney MS, Steele A, Newman J (2024). Digital endpoints in clinical trials: emerging themes from a multi-stakeholder Knowledge Exchange event. Trials.

[14] Prisco G, Pirozzi MA, Santone A (2025). Validity of Wearable Inertial Sensors for Gait Analysis: A Systematic Review. Diagnostics.

[15] Peters MDJ, Marnie C, Tricco AC (2020). Updated methodological guidance for the conduct of scoping reviews. *JBI Evid Synth*.

[16] Tricco AC, Lillie E, Zarin W (2018). PRISMA Extension for Scoping Reviews (PRISMA-ScR): Checklist and Explanation. Ann Intern Med.

[17] Prinsen CAC, Mokkink LB, Bouter LM (2018). COSMIN guideline for systematic reviews of patient-reported outcome measures. Qual Life Res.

