# Supplementary material for: Digital endpoints as outcome measures in orthopaedic care and their psychometric properties: a scoping review protocol
Source: BMJ Open. 2026 Nov 3;16(11):e117633. doi: 10.1136/bmjopen-2026-117633 (PMC13735298; doi:10.1136/bmjopen-2026-117633)
Supplement: Supplementary file 1 [file bmjopen-16-11-s001.pdf]

Appendices

Appendix I: Search strategy

Search Strategy

Databases (from 2005 to December 2025)

- Embase (4796)
- MEDLINE (PubMed) (4729)
- CINAHL (762)
- IEEE Xplore (for engineering/sensor papers) (372)
- Cochrane Library (679)
- Web of Science (1,371)

Grey Literature

- Not included

Search terms

Digital endpoints

Digital health	Activity tracker OR activity monitor or fitness tracker
Digital endpoint	smart* implant
wearable*	gyroscop*
acceleromet*	magnetomet*
Inertial measurement unit*	pedometer*
IMU	Smart watch or band or device or wearable
Smartphone OR mobile phone or cell phone	Smart implant
Mobile health	Fitness tracker
mHealth	

sensor	
Remote monitoring	
Wearable electronic devices	
Wearable devic*	
telerehab*	

Table 3. Full MEDLINE (Ovid) search syntax.

Orthopaedic surgery and care

orthop?edic* OR orthopaedic* OR orthopedic*	
Spine	
Cervical (spine or spinal or vertebra or radiculopath or myelopath* or disc or fusion)	
Spinal Injuries or Spinal Fusion or Diskectomy or ("spinal injur*" or "spinal fusion" or "lumbar fusion" or discectom* or "spinal fracture*" or "nerve root*" or "low back pain" or sciatica	
Hip or Hip Joint or (hip or hips or THR or THA)	
Knee or knee joint or TKR or TKA	
Ankle	
Elbow	
Wrist	
shoulder OR “shoulder Joint” OR shoulder	
Fractures	
Arthroplasty	
Arthroscopy	
osteoarthritis	

EMBASE - 4796

Embase <1974 to 2025 December 11>

1 (digital adj1 health).ti,ab,kw. or exp Digital Health/ 15930

2 ((mobile adj health) or mhealth).ti,ab,kw. 18458

3 (smartphone* or "mobile phone*" or cellphone*).ti,ab,kw. or exp Smartphones/ 74086

4 (wearable* or "wearable devic*" or "wearable electronic device*" or "wearable technology").ti,ab,kw. or exp Wearable Electronic Devices/ 65149

5 ((activity adj2 (track* or monitor*)) or "activity tracker*" or "activity monitor*" or "fitness tracker*").ti,ab,kw. 27047

6 ((digital adj2 endpoint*) or "digital endpoint*").ti,ab,kw. 154

7 (acceleromet* or gyroscop* or magnetomet* or pedometer* or "inertial measurement unit*" or IMU).ti,ab,kw. 54162

8 (sensor* adj3 (wearable* or device* or IMU or acceleromet* or gyroscop*)).ti,ab,kw. 19620

9 (telerehab* or telerehabilitat*).ti,ab,kw. 4451

10 ("smart implant*" or (smart adj2 implant*)).ti,ab,kw. 298

11 (smart adj3 (watch* or band* or wearable* or device*)).ti,ab,kw. 6133

12 (remote adj2 monitor*).ti,ab,kw. 13786

13 or/1-12 241244

14 exp Orthopedics/ or exp Orthopedic Procedures/ or orthop?edic*.ti,ab,kw. 799787

15 exp Spine/ or (spine or spinal* or lumbar* or "thoracic spine" or "thoracolumbar").ti,ab,kw. 781038

16 (cervical adj3 (spine or spinal or vertebra or radiculopath or myelopath* or disc or fusion)).ti,ab,kw. 68386

17 exp Spinal Injuries/ or exp Spinal Fusion/ or exp Diskectomy/ or ("spinal injur*" or "spinal fusion" or "lumbar fusion" or discectom* or "spinal fracture*" or "nerve root*" or "low back pain" or sciatica).ti,ab,kw. 212779

18 exp Hip/ or exp Hip Joint/ or (hip or hips or THR or THA).ti,ab,kw. 364158

19 exp Knee/ or exp Knee Joint/ or (knee or knees or "total knee" or TKR or TKA).ti,ab,kw. 300855

20 exp Ankle/ or exp Ankle Joint/ or ankle*.ti,ab,kw. 127600

21 exp Elbow/ or exp Elbow Joint/ or elbow*.ti,ab,kw. 65558

22 exp Wrist/ or exp Wrist Joint/ or wrist*.ti,ab,kw. 88355

23 exp Shoulder/ or exp Shoulder Joint/ or shoulder*.ti,ab,kw. or ("rotator cuff" or (rotator adj1 cuff)).ti,ab,kw. 182990

24 exp Fractures, Bone/ or (fracture* or "spinal fracture*" or (orthop adj3 trauma)).ti,ab,kw. 530925

25 exp Arthroplasty/ or arthroplast.ti,ab,kw. or ("joint replacement" or (joint adj2 replacement*)).ti,ab,kw. 155909

26 exp Arthroscopy/ or arthroscop*.ti,ab,kw. 73718

27 ((osteoarthritis or tendinopath* or menisc* or ACL) and (surg* or operat* or procedur* or arthroplast* or arthroscop* or replacement* or reconstruction* or repair* or osteotom* or fusion)).ti,ab,kw. 98588

28 14 or 15 or 17 or 18 or 19 or 20 or 21 or 22 or 23 or 24 or 25 or 26 or 27 2309441

29 "Reproducibility of Results"/ or "Validation Study"/ or (validity or validat* or "construct validity" or "criterion validity" or "content validity").ti,ab,kw. 1870137

30 (reliability or reproducib* or repeatab*).ti,ab,kw. 643351

31 Psychometrics/ or (psychometr* or clinimetr*).ti,ab,kw. 131418

32 ("measurement propert*" or (measurement adj2 propert*)).ti,ab,kw. 6674

33 (responsiveness or (change adj2 (score* or detect* or measur*))).ti,ab,kw. 214550

34 ("minimal clinically important difference" or "minimally clinically important difference" or "minimal important difference" or MCID or MIC).ti,ab,kw. 88728

35 ("minimal detectable change" or "minimum detectable change" or MDC).ti,ab,kw. 9863

36 (ePRO or ePROM or "electronic patient-reported outcome*" or "electronic PROM*" or "electronic PRO*").ti,ab,kw. 20278

37 ((electronic* or digital*) adj3 (PRO or PROM or outcome* or endpoint* or measure* or questionnaire* or instrument*)).ti,ab,kw. 31506

38 29 or 30 or 31 or 32 or 33 or 34 or 35 or 36 or 37 2588223

39 13 and 28 and 38 6074

40 exp Neoplasms/ or (cancer* or tumor* or tumour* or oncology or breast or lung or prostate or colorectal or melanoma).ti. 7203045

41 exp Obstetrics/ or exp Pregnancy/ or (pregnan* or obstetrics or gynecolog* or uteri or womb or birth or trimester).ti,kw. 1109042

42 exp Parkinson Disease/ or exp Stroke/ or exp Multiple Sclerosis/ or exp Alzheimer Disease/ or exp Cerebral Palsy/ or (parkinson or stroke or CVA or "cerebral palsy" or "multiple sclerosis" or MS or alzheimer or dementia or epilepsy or "brain injury").ti. 1566297

43 ("medical education" or "surgical education" or curriculum or "medical student" or residency or internship).ti,kw. 89434

44 or/40-43 9744663

45 39 not 44 5121

46 limit 45 to (human and yr="2005 -Current") 4796

PUBMED - 4729

#1

(

("Digital Health"[MeSH] OR ("digital"[tiab] AND "health"[tiab]) OR "digital health"[tiab]) OR (("mobile"[tiab] AND "health"[tiab]) OR "mobile health"[tiab] OR mhealth[tiab]) OR ("Smartphones"[MeSH] OR smartphone*[tiab] OR (mobile[tiab] AND phone*[tiab]) OR "mobile phone"[tiab] OR cellphone*[tiab]) OR ("Wearable Electronic Devices"[MeSH] OR wearable*[tiab] OR (wearable[tiab] AND devic*[tiab]) OR "wearable device"[tiab] OR (wearable[tiab] AND electronic[tiab]

AND device*[tiab]) OR "wearable electronic device"[tiab] OR "wearable technology"[tiab]) OR ((activity[tiab] AND (track*[tiab] OR monitor*[tiab])) OR "activity tracker"[tiab] OR "activity trackers"[tiab] OR "activity monitor"[tiab] OR "activity monitors"[tiab] OR "fitness tracker"[tiab] OR "fitness trackers"[tiab]) OR (("digital"[tiab] AND endpoint*[tiab]) OR "digital endpoint"[tiab] OR "digital endpoints"[tiab]) OR (acceleromet*[tiab] OR gyroscop*[tiab] OR magnetomet*[tiab] OR pedometer*[tiab] OR "inertial measurement unit"[tiab] OR "inertial measurement units"[tiab] OR IMU[tiab]) OR (sensor*[tiab] AND (wearable*[tiab] OR device*[tiab] OR IMU[tiab] OR acceleromet*[tiab] OR gyroscop*[tiab])) OR (telerehab*[tiab] OR telerehabilitat*[tiab]) OR ("smart implant"[tiab] OR "smart implants"[tiab] OR (smart[tiab] AND implant*[tiab])) OR (smartwatch*[tiab] OR (smart[tiab] AND (watch*[tiab] OR band*[tiab] OR wearable*[tiab] OR device*[tiab]))) OR ("remote monitoring"[tiab] OR (remote[tiab] AND monitor*[tiab]))

)

#2

(

"Orthopedics"[MeSH] OR "Orthopedic Procedures"[MeSH] OR orthopaedic*[tiab] OR orthopedic*[tiab] OR "Spine"[MeSH] OR spine[tiab] OR spinal*[tiab] OR lumbar*[tiab] OR "thoracic spine"[tiab] OR thoracolumbar[tiab] OR (cervical[tiab] AND (spine[tiab] OR spinal[tiab] OR vertebra*[tiab] OR radiculopath*[tiab] OR myelopath*[tiab] OR disc*[tiab] OR fusion[tiab])) OR "Spinal Injuries"[MeSH] OR "Spinal Fusion"[MeSH] OR "Diskectomy"[MeSH] OR "spinal injur*"[tiab] OR "spinal fusion"[tiab] OR "lumbar fusion"[tiab] OR discectom*[tiab] OR "spinal fracture*"[tiab] OR "nerve root*"[tiab] OR "low back pain"[tiab] OR sciatica[tiab] OR "Hip"[MeSH] OR "Hip Joint"[MeSH] OR hip[tiab] OR hips[tiab] OR THR[tiab] OR THA[tiab] OR "Knee"[MeSH] OR "Knee Joint"[MeSH] OR knee[tiab] OR knees[tiab] OR "total knee"[tiab] OR TKR[tiab] OR TKA[tiab] OR "Ankle"[MeSH] OR "Ankle Joint"[MeSH] OR ankle*[tiab] OR "Elbow"[MeSH] OR "Elbow Joint"[MeSH] OR elbow*[tiab] OR "Wrist"[MeSH] OR "Wrist Joint"[MeSH] OR wrist*[tiab] OR "Shoulder"[MeSH] OR "Shoulder Joint"[MeSH] OR shoulder*[tiab] OR "rotator cuff"[tiab] OR (rotator[tiab] AND cuff[tiab]) OR "Fractures, Bone"[MeSH] OR fracture*[tiab] OR "spinal fracture*"[tiab] OR (orthop*[tiab] AND trauma[tiab]) OR "Arthroplasty"[MeSH] OR arthroplast*[tiab] OR "joint replacement"[tiab] OR (joint[tiab] AND replacement*[tiab]) OR "Arthroscopy"[MeSH] OR arthroscop*[tiab] OR ((osteoarthritis[tiab] OR tendinopath*[tiab] OR menisc*[tiab] OR ACL[tiab]) AND (surg*[tiab] OR operat*[tiab] OR procedur*[tiab] OR arthroplast*[tiab] OR arthroscop*[tiab] OR replacement*[tiab] OR reconstruction*[tiab] OR repair*[tiab] OR osteotom*[tiab] OR fusion[tiab]))

)

#3

(

"Reproducibility of Results"[MeSH] OR "Validation Study"[Publication Type] OR validity[tiab] OR validat*[tiab] OR "construct validity"[tiab] OR "criterion validity"[tiab] OR "content validity"[tiab] OR reliability[tiab] OR reproducib*[tiab] OR repeatab*[tiab] OR "Psychometrics"[MeSH] OR psychometr*[tiab] OR clinimetr*[tiab] OR "measurement propert*"[tiab] OR (measurement[tiab] AND propert*[tiab]) OR responsiveness[tiab] OR (change[tiab] AND (score*[tiab] OR detect*[tiab] OR measur*[tiab])) OR "minimal clinically important difference"[tiab] OR "minimally clinically important difference"[tiab] OR "minimal important difference"[tiab] OR MCID[tiab] OR MIC[tiab] OR "minimal detectable change"[tiab] OR "minimum detectable change"[tiab] OR MDC[tiab] OR ePRO[tiab] OR ePROM[tiab] OR "electronic patient-reported outcome*"[tiab] OR "electronic PROM*"[tiab] OR "electronic PRO*"[tiab] OR ((electronic*[tiab] OR digital*[tiab]) AND (PRO[tiab] OR PROM[tiab] OR outcome*[tiab] OR endpoint*[tiab] OR measure*[tiab] OR questionnaire*[tiab] OR instrument*[tiab]))

)

#4

#1 AND #2 AND #3

#5

(

"Neoplasms"[MeSH] OR cancer*[ti] OR tumor*[ti] OR tumour*[ti] OR oncology[ti] OR breast[ti] OR lung[ti] OR prostate[ti] OR colorectal[ti] OR melanoma[ti] OR "Obstetrics"[MeSH] OR "Pregnancy"[MeSH] OR pregnan*[ti] OR obstetrics[ti] OR gynecolog*[ti] OR uteri[ti] OR womb[ti] OR birth[ti] OR trimester[ti] OR "Parkinson Disease"[MeSH] OR "Stroke"[MeSH] OR "Multiple Sclerosis"[MeSH] OR "Alzheimer Disease"[MeSH] OR "Cerebral Palsy"[MeSH] OR parkinson[ti] OR stroke[ti] OR CVA[ti] OR "cerebral palsy"[ti] OR "multiple sclerosis"[ti] OR MS[ti] OR alzheimer[ti] OR dementia[ti] OR epilepsy[ti] OR "brain injury"[ti] OR "Education, Medical"[MeSH] OR "medical education"[ti] OR "surgical education"[ti] OR curriculum[ti] OR "medical student"[ti] OR residency[ti] OR internship[ti]

)

#6

(#4 NOT #5) AND "humans"[MeSH Terms] AND ("2005/01/01"[dp] : "2500"[dp])

Results: 4729

Cochrane Library - 679

#1

(

[mh "Digital Health"] OR [mh "Smartphone"] OR [mh "Wearable Electronic Devices"] OR (digital NEAR/1 health):ti,ab,kw OR ((mobile NEAR/1 health) OR mhealth):ti,ab,kw OR (smartphone* OR (mobile NEXT phone*) OR cellphone*):ti,ab,kw OR (wearable* OR (wearable NEXT devic*) OR (wearable NEXT electronic NEXT device*) OR (wearable NEXT technolog*)):ti,ab,kw OR ((activity NEAR/2 (track* OR monitor*)) OR (activity NEXT tracker*) OR (activity NEXT monitor*) OR (fitness NEXT tracker*)):ti,ab,kw OR ((digital NEAR/2 endpoint*) OR (digital NEXT endpoint*)):ti,ab,kw OR (acceleromet* OR gyroscop* OR magnetomet* OR pedometer* OR (inertial NEXT measurement NEXT unit*) OR IMU):ti,ab,kw OR (sensor* NEAR/3 (wearable* OR device* OR IMU OR acceleromet* OR gyroscop*)):ti,ab,kw OR (telerehab* OR telerehabilitat*):ti,ab,kw OR ((smart NEXT implant*) OR (smart NEAR/2 implant*)):ti,ab,kw OR (smart NEAR/3 (watch* OR band* OR wearable* OR device*)):ti,ab,kw OR (remote NEAR/2 monitor*):ti,ab,kw

)

#2

(

[mh "Orthopedics"] OR [mh "Orthopedic Procedures"] OR [mh "Spine"] OR [mh "Spinal Injuries"] OR [mh "Spinal Fusion"] OR [mh "Diskectomy"] OR [mh "Hip"] OR [mh "Hip Joint"] OR [mh "Knee"] OR [mh "Knee Joint"] OR [mh "Ankle"] OR [mh "Ankle Joint"] OR [mh "Elbow"] OR [mh "Elbow Joint"] OR [mh "Wrist"] OR [mh "Wrist Joint"] OR [mh "Shoulder"] OR [mh "Shoulder Joint"] OR [mh "Fractures, Bone"] OR [mh "Arthroplasty"] OR [mh "Arthroscopy"] OR orthop?edic*:ti,ab,kw OR (spine OR spinal* OR lumbar* OR (thoracic NEXT spine) OR thoracolumbar):ti,ab,kw OR (cervical NEAR/3 (spine OR spinal OR vertebra OR radiculopath OR myelopath* OR disc OR fusion)):ti,ab,kw OR ((spinal NEXT injur*) OR (spinal NEXT fusion) OR (lumbar NEXT fusion) OR discectom* OR (spinal NEXT fracture*) OR (nerve NEXT root*) OR (low NEXT back NEXT pain) OR sciatica):ti,ab,kw OR (hip OR hips OR THR OR THA):ti,ab,kw OR (knee OR knees OR (total NEXT knee) OR TKR OR TKA):ti,ab,kw OR ankle*:ti,ab,kw

OR elbow*:ti,ab,kw OR wrist*:ti,ab,kw OR (shoulder* OR (rotator NEXT cuff) OR (rotator NEAR/1 cuff)):ti,ab,kw OR (fracture* OR (orthop NEAR/3 trauma)):ti,ab,kw OR arthroplast*:ti,ab,kw OR arthroscop*:ti,ab,kw OR ((osteoarthritis OR tendinopath* OR menisc* OR ACL) AND (surg* OR operat* OR procedur* OR arthroplast* OR arthroscop* OR replacement* OR reconstruction* OR repair* OR osteotom* OR fusion)):ti,ab,kw
)

#3
(
[mh "Reproducibility of Results"] OR [mh "Validation Study"] OR [mh "Psychometrics"] OR (validity OR validat* OR reliability OR reproducib* OR repeatab* OR psychometr* OR clinimetr*):ti,ab,kw OR (measurement NEAR/2 propert*):ti,ab,kw OR (responsiveness OR (change NEAR/2 (score* OR detect* OR measur*))):ti,ab,kw OR ((minimal NEXT clinically NEXT important NEXT difference) OR (minimally NEXT clinically NEXT important NEXT difference) OR (minimal NEXT important NEXT difference) OR MCID OR MIC):ti,ab,kw OR ((minimal NEXT detectable NEXT change) OR (minimum NEXT detectable NEXT change) OR MDC):ti,ab,kw OR (ePRO OR ePROM OR (electronic NEXT patient-reported NEXT outcome*) OR (electronic NEXT PROM*) OR (electronic NEXT PRO*)):ti,ab,kw OR ((electronic* OR digital*) NEAR/3 (PRO OR PROM OR outcome* OR endpoint* OR measure* OR questionnaire* OR instrument*)):ti,ab,kw
)

#4 #1 AND #2 AND #3

#5
(
[mh "Neoplasms"] OR [mh "Pregnancy"] OR [mh "Obstetrics"] OR [mh "Parkinson Disease"] OR [mh "Stroke"] OR [mh "Multiple Sclerosis"] OR [mh "Alzheimer Disease"] OR [mh "Cerebral Palsy"] OR (cancer* OR tumor* OR tumour* OR oncology OR breast OR lung OR prostate OR colorectal OR melanoma):ti OR (pregnan* OR obstetrics OR gynecolog* OR uteri OR womb OR birth OR trimester):ti,kw OR (parkinson OR stroke OR CVA OR (cerebral NEXT palsy) OR (multiple NEXT sclerosis) OR MS OR alzheimer OR dementia OR epilepsy OR (brain NEXT injury)):ti OR ((medical NEXT education) OR (surgical NEXT education) OR curriculum OR (medical NEXT student) OR residency OR internship):ti,kw
)

#6

#4 NOT #5

Restrict to 2005 onwards

RESULTS: 679

IEEE Xplore - 372

(

"digital health" OR (digital NEAR/1 health) OR "mobile health" OR mhealth OR smartphone OR "mobile phone" OR "cell phone" OR cellphone OR wearable* OR "wearable device" OR "wearable electronic device" OR "wearable technology" OR "activity tracker" OR "activity monitor" OR "fitness tracker" OR (activity NEAR/2 (track OR tracking OR monitor OR monitoring)) OR "digital endpoint" OR (digital NEAR/2 endpoint) OR acceleromet* OR gyroscop* OR magnetomet* OR pedometer* OR

"inertial measurement unit" OR IMU OR (sensor* NEAR/3 (wearable OR device OR IMU OR accelerometer OR gyroscope)) OR telerehab* OR telerehabilitation OR "smart implant" OR (smart NEAR/2 implant) OR (smart NEAR/3 (watch OR watches OR band OR bands OR wearable OR device)) OR (remote NEAR/2 monitor*)
)

AND

(
orthopaedic OR orthopaedics OR orthopedic OR orthopedics OR musculoskeletal OR MSK OR spine OR spinal OR lumbar OR thoracic OR thoracolumbar OR cervical OR "spinal injury" OR "spinal fusion" OR "lumbar fusion" OR discectomy OR "low back pain" OR sciatica OR "nerve root" OR hip OR THA OR THR OR knee OR TKA OR TKR OR ankle OR elbow OR wrist OR shoulder OR "rotator cuff" OR fracture OR arthroplasty OR arthroscopy OR "joint replacement" OR osteoarthritis OR tendinopathy OR meniscus OR ACL
)

AND

(
surgery OR surgical OR operation OR operative OR procedure OR replacement OR reconstruction OR repair OR fusion OR osteotomy
)

AND

(
validity OR validation OR "construct validity" OR "criterion validity" OR "content validity" OR reliability OR reproducibility OR repeatability OR psychometrics OR psychometric OR clinimetrics OR clinimetric OR "measurement property" OR "measurement properties" OR responsiveness OR (change NEAR/2 (score OR scores OR detect OR detection OR measure OR measurement)) OR "minimal clinically important difference" OR "minimally clinically important difference" OR "minimal important difference" OR MCID OR MIC OR "minimal detectable change" OR "minimum detectable change" OR MDC OR ePRO OR ePROM OR "electronic patient reported outcome" OR "electronic patient-reported

outcome" OR "electronic PROM" OR "electronic PRO" OR ((electronic OR digital) NEAR/3 (PRO OR PROM OR outcome OR outcomes OR endpoint OR endpoints OR measure OR measures OR questionnaire OR questionnaires OR instrument OR instruments))
)

NOT

(
cancer OR tumor OR tumour OR oncology OR breast OR lung OR prostate OR colorectal OR melanoma OR pregnancy OR obstetrics OR gynecology OR gynaecology OR uteri OR uterus OR womb OR birth OR trimester OR parkinson OR stroke OR CVA OR "cerebral palsy" OR "multiple sclerosis" OR MS OR alzheimer OR dementia OR epilepsy OR "brain injury" OR "medical education" OR "surgical education" OR curriculum OR "medical student" OR residency OR internship
)

Filter 2005 - 2026

RESULTS: 372

Web of science - 1371

#1 TS=(

(digital NEAR/1 health) OR (mobile NEAR/1 health) OR mhealth OR smartphone* OR (mobile NEAR/1 phone*) OR cellphone* OR wearable* OR (wearable NEAR/1 devic*) OR (wearable NEAR/2 (electronic NEAR/1 devic*)) OR (wearable NEAR/1 technolog*) OR (activity NEAR/2 (track* OR monitor*)) OR (activity NEAR/1 tracker*) OR (activity NEAR/1 monitor*) OR (fitness NEAR/1 tracker*) OR (digital NEAR/2 endpoint*) OR (digital NEAR/1 endpoint*) OR acceleromet* OR gyroscop* OR magnetomet* OR pedometer* OR (inertial NEAR/1 measurement NEAR/1 unit*) OR IMU OR (sensor* NEAR/3 (wearable* OR device* OR IMU OR acceleromet* OR gyroscop*)) OR telerehab* OR telerehabilitat* OR (smart NEAR/1 implant*) OR (smart NEAR/3 (watch* OR band* OR wearable* OR device*)) OR (remote NEAR/2 monitor*)

)

#2 TS=(

orthop?edic* OR spine OR spinal* OR lumbar* OR ("thoracic spine") OR thoracolumbar OR (cervical NEAR/3 (spine OR spinal OR vertebra OR radiculopath* OR myelopath* OR disc OR fusion)) OR (spinal NEAR/1 injur*) OR (spinal NEAR/1 fusion) OR (lumbar NEAR/1 fusion) OR discectom* OR (spinal NEAR/1 fracture*) OR (nerve NEAR/1 root*) OR (low NEAR/1 back NEAR/1 pain) OR sciatica OR hip OR hips OR THR OR THA OR knee OR knees OR (total NEAR/1 knee) OR TKR OR TKA OR ankle* OR elbow* OR wrist* OR shoulder* OR (rotator NEAR/1 cuff) OR fracture* OR (orthop* NEAR/3 trauma) OR arthroplast* OR (joint NEAR/2 replacement*) OR arthroscop* OR ((osteoarthritis OR tendinopath* OR

menisc* OR ACL) AND (surg* OR operat* OR procedur* OR arthroplast* OR arthroscop* OR replacement* OR reconstruction* OR repair* OR osteotom* OR fusion))
)

#3 TS=(
surg* OR operat* OR procedure* OR perioperat* OR intraoperat* OR postoperative OR postoperat* OR arthroplast* OR arthroscop* OR replacement* OR "joint replacement" OR reconstruction* OR repair* OR revision* OR osteotom* OR fusion OR discectom* OR diskectom* OR laminectom* OR decompression OR "open reduction" OR "internal fixation" OR ORIF OR fixation
)

#4 TS=(
validity OR validat* OR (construct NEAR/1 validity) OR (criterion NEAR/1 validity) OR (content NEAR/1 validity) OR reliability OR reproducib* OR repeatab* OR psychometr* OR clinimetr* OR (measurement NEAR/2 propert*) OR responsiveness OR (change NEAR/2 (score* OR detect* OR measur*)) OR ("minimal clinically important difference") OR ("minimally clinically important difference") OR ("minimal important difference") OR MCID OR MIC OR ("minimal detectable change") OR ("minimum detectable change") OR MDC OR ePRO OR ePROM OR ((electronic* OR digital*) NEAR/3 (PRO OR PROM OR outcome* OR endpoint* OR measure* OR questionnaire* OR instrument*)) OR ((electronic NEAR/2 patient NEAR/2 reported NEAR/2 outcome*) OR ("electronic patient-reported outcome*") OR ("electronic PROM*") OR ("electronic PRO*"))
)

#5 (TI=(cancer* OR tumor* OR tumour* OR oncology OR breast OR lung OR prostate OR colorectal OR melanoma) OR (TI=(pregnan* OR obstetrics OR gynecolog* OR uteri OR womb OR birth OR trimester) OR AK=(pregnan* OR obstetrics OR gynecolog* OR uteri OR womb OR birth OR trimester)) OR TI=(parkinson* OR stroke OR CVA OR "cerebral palsy" OR "multiple sclerosis" OR MS OR alzheimer* OR dementia OR epilepsy OR "brain injury") OR (TI=("medical education" OR "surgical education" OR curriculum OR "medical student" OR residency OR internship) OR AK=("medical education" OR "surgical education" OR curriculum OR "medical student" OR residency OR internship))
)

#6 (#1 AND #2 AND #3 AND #4) NOT #5

#7 TS=(mouse OR mice OR rat OR rats OR murine OR rabbit* OR porcine OR swine OR pig* OR canine OR dog OR dogs OR feline OR cat OR cats OR bovine OR sheep OR ovine OR animal*)

#8 TS=(nanoparticle* OR graphene OR "carbon nanotube*" OR synthesis OR catalyst* OR photocatal* OR polymer* OR "thin film*" OR semiconductor* OR dielectric* OR spectroscop* OR electrochem*)

#9 #6 NOT #7

#10 #9 NOT #8

Restrict to articles and >2005

RESULTS: 1371

CINHL - 762

(

(MH "Digital Health") OR (MH "Smartphones+") OR (MH "Wearable Technology+") OR TI((digital N1 health) OR (mobile N1 health) OR mhealth OR smartphone* OR "mobile phone*" OR cellphone* OR wearable* OR "wearable devic*" OR "wearable electronic device*" OR "wearable technology" OR (activity N2 (track* OR monitor*)) OR "activity tracker*" OR "activity monitor*" OR "fitness tracker*" OR (digital N2 endpoint*) OR "digital endpoint*" OR acceleromet* OR gyroscop* OR magnetomet* OR pedometer* OR "inertial measurement unit*" OR IMU OR (sensor* N3 (wearable* OR device* OR IMU OR acceleromet* OR gyroscop*)) OR telerehab* OR telerehabilitat* OR "smart implant*" OR (smart N2 implant*) OR (smart N3 (watch* OR band* OR wearable* OR device*)) OR (remote N2 monitor*)) OR AB((digital N1 health) OR (mobile N1 health) OR mhealth OR smartphone* OR "mobile phone*" OR cellphone* OR wearable* OR "wearable devic*" OR "wearable electronic device*" OR "wearable technology" OR (activity N2 (track* OR monitor*)) OR "activity tracker*" OR "activity monitor*" OR "fitness tracker*" OR (digital N2 endpoint*) OR "digital endpoint*" OR acceleromet* OR gyroscop* OR magnetomet* OR pedometer* OR "inertial measurement unit*" OR IMU OR (sensor*

N3 (wearable* OR device* OR IMU OR acceleromet* OR gyroscop*)) OR telerehab* OR telerehabilitat* OR "smart implant*" OR (smart N2 implant*) OR (smart N3 (watch* OR band* OR wearable* OR device*)) OR (remote N2 monitor*)

)

)

(

(MH "Orthopedics+") OR (MH "Orthopedic Procedures+") OR (MH "Spine+") OR (MH "Spinal Injuries+") OR (MH "Spinal Fusion+") OR (MH "Diskectomy+") OR (MH "Hip+") OR (MH "Hip Joint+") OR (MH "Knee+") OR (MH "Knee Joint+") OR (MH "Ankle+") OR (MH "Ankle Joint+") OR (MH "Elbow+") OR (MH "Elbow Joint+") OR (MH "Wrist+") OR (MH "Wrist Joint+") OR (MH "Shoulder+") OR (MH "Shoulder Joint+") OR (MH "Fractures, Bone+") OR (MH "Arthroplasty+") OR (MH "Arthroscopy+") OR TI(orthop?edic* OR spine OR spinal* OR lumbar* OR "thoracic spine" OR "thoracolumbar" OR (cervical N3 (spine OR spinal OR vertebra OR radiculopath OR myelopath* OR disc OR fusion)) OR "spinal injur*" OR "spinal fusion" OR "lumbar fusion" OR discectom* OR "spinal fracture*" OR "nerve root*" OR "low back pain" OR sciatica OR hip OR hips OR THR OR THA OR knee OR knees OR "total knee" OR TKR OR TKA OR ankle* OR elbow* OR wrist* OR shoulder* OR "rotator cuff" OR (rotator N1 cuff) OR fracture* OR (orthop N3 trauma) OR arthroplast* OR "joint replacement" OR (joint N2 replacement*) OR arthroscop* OR ((osteoarthritis OR tendinopath* OR menisc* OR ACL) AND (surg* OR operat* OR procedur* OR arthroplast* OR arthroscop* OR replacement* OR reconstruction* OR repair* OR osteotom* OR fusion))) OR AB(orthop?edic* OR spine OR spinal* OR lumbar* OR "thoracic spine" OR "thoracolumbar" OR (cervical N3 (spine OR spinal OR vertebra OR radiculopath OR myelopath* OR disc OR fusion)) OR "spinal injur*" OR "spinal fusion" OR "lumbar fusion" OR discectom* OR "spinal fracture*" OR "nerve root*" OR "low back pain" OR sciatica OR hip OR hips OR THR OR THA OR knee OR knees OR "total knee" OR TKR OR TKA OR ankle* OR elbow* OR wrist* OR shoulder* OR "rotator cuff" OR (rotator N1 cuff) OR fracture* OR (orthop N3 trauma) OR arthroplast* OR "joint replacement" OR (joint N2 replacement*) OR arthroscop* OR ((osteoarthritis OR tendinopath* OR menisc* OR ACL) AND (surg* OR operat* OR procedur* OR arthroplast* OR arthroscop* OR replacement* OR reconstruction* OR repair* OR osteotom* OR fusion)))

)

(

(MH "Reproducibility of Results") OR (MH "Validation Studies") OR (MH "Psychometrics+") OR TI(validity OR validat* OR "construct validity" OR "criterion validity" OR "content validity" OR reliability OR reproducib* OR repeatab* OR psychometr* OR clinimetr* OR "measurement propert*" OR (measurement N2 propert*) OR responsiveness OR (change N2 (score* OR detect* OR measur*)) OR "minimal clinically important difference" OR "minimally clinically important difference" OR "minimal important difference" OR MCID OR MIC OR "minimal detectable change" OR "minimum detectable change" OR MDC OR ePRO OR ePROM OR "electronic patient-reported outcome*" OR "electronic PROM*" OR "electronic PRO*" OR ((electronic* OR digital*) N3 (PRO OR PROM OR outcome* OR endpoint* OR measure* OR questionnaire* OR instrument*)))
OR AB(validity OR validat* OR "construct validity" OR "criterion validity" OR "content validity"
OR reliability OR reproducib* OR repeatab* OR psychometr* OR clinimetr* OR "measurement propert*" OR (measurement N2 propert*) OR responsiveness OR (change N2 (score* OR detect* OR measur*)) OR "minimal clinically important difference" OR "minimally clinically important difference" OR "minimal important difference" OR MCID OR MIC OR "minimal detectable change" OR "minimum detectable change" OR MDC OR ePRO OR ePROM OR "electronic patient-reported outcome*" OR "electronic PROM*" OR "electronic PRO*" OR ((electronic* OR digital*) N3 (PRO OR PROM OR outcome* OR endpoint* OR measure* OR questionnaire* OR instrument*)))
)

S1 AND S2 AND S3

(
(MH "Neoplasms+") OR (MH "Pregnancy+") OR (MH "Obstetrics+") OR (MH "Parkinson Disease+") OR (MH "Stroke+") OR (MH "Multiple Sclerosis+") OR (MH "Alzheimer's Disease+") OR (MH "Cerebral Palsy+") OR TI(cancer* OR tumor* OR tumour* OR oncology OR breast OR lung OR prostate OR colorectal OR melanoma OR pregnan* OR obstetrics OR gynecolog* OR uteri OR womb OR birth OR trimester OR parkinson OR stroke OR CVA OR "cerebral palsy" OR "multiple sclerosis" OR MS OR alzheimer OR dementia OR epilepsy OR "brain injury" OR "medical education" OR "surgical education" OR curriculum OR "medical student" OR residency OR internship)
)

S4 NOT S5

Results: 762
