# Supplementary material for: Digital endpoints as outcome measures in orthopaedic care and their psychometric properties: a scoping review protocol
Source: BMJ Open. 2026 Nov 3;16(11):e117633. doi: 10.1136/bmjopen-2026-117633 (PMC13735298; doi:10.1136/bmjopen-2026-117633)
Supplement: Supplementary file 2 [file bmjopen-2026-117633.reviewer_comments.pdf]

PEER REVIEW HISTORY

BMJ Open publishes all reviews undertaken for accepted manuscripts. Reviewers are asked to complete a checklist review form and are provided with free text boxes to elaborate on their assessment. These free text comments are reproduced below.

ARTICLE DETAILS

Title (Provisional)

Digital Endpoints as Outcome Measures in Orthopaedic Care and Their Psychometric Properties: A Scoping Review Protocol

Authors

Fakak, Reem; Aslam, Sultan; Davies, Benjamin; Hadi, Saif; Gharooni, Ali

---

VERSION 1 - REVIEW

**Reviewer**	**1**
**Name**	**Nicolson, Philippa**
**Affiliation**	**University of Oxford, Nuffield Department of Orthopaedics, Rheumatology and Musculoskeletal Sciences**
**Date**	**16-Jul-2026**
**COI**	**None**

Thank you for the opportunity to review this manuscript, which reports the protocol for a scoping review of digital endpoints as outcome measures in orthopaedic

surgery and musculoskeletal care and their psychometric properties.

I have one major concern about the manuscript - terminology used throughout is inconsistent meaning it is unclear what the focus of the review is. The title states ‘orthopaedic and musculoskeletal care’ but throughout the manuscript often only ‘orthopaedic ‘is used e.g. Line 29, Line 45, Strengths and Limitations. Introduction Lines 5, 36, 45, Table 1. Concerningly the search terms do not include musculoskeletal care or related terms, so this area will not be included.

Introduction

Why a scoping review not a systematic review? Important to justify this and how the two methods differ.

Review question

States only ‘What digital endpoints have been evaluated for their psychometric properties in orthopaedic care?’ – this would suggest the authors are only interested in which endpoints

have been evaluated, not what the psychometric properties are, and again no mention of musculoskeletal care as well as orthopaedic?

Page 5 Participants

Line 23 describes eligible participants, but then Line 33 also does – this should all be together

Line 46: 'populations in which findings are not clearly transferable to orthopaedic care' – what do the authors mean by this?

Page 6 Concept

It would be helpful to the reader to expand on the exact psychometric properties of interest

Page 6 Context

The authors state 'The focus is on measures intended for practical outcome assessment beyond highly controlled laboratory settings' – what do you mean by this?

Appendix 1 Search strategy

There are no musculoskeletal care search terms? The current search is limited to orthopaedic terms – if this has already been run then the manuscript needs to reflect that only orthopaedic conditions are of interest.

VERSION 1 - AUTHOR RESPONSE

Responses to Reviewer 1

Comment 1: *I have one major concern about the manuscript - terminology used throughout is inconsistent meaning it is unclear what the focus of the review is. The title states 'orthopaedic and musculoskeletal care' but throughout the manuscript often only 'orthopaedic 'is used. Concerningly the search terms do not include musculoskeletal care or related terms, so this area will not be included.*
Response: We sincerely thank the reviewer for highlighting this discrepancy. We completely agree that our true focus, as reflected by our search strategy, is strictly orthopaedic care. To resolve this inconsistency, we have removed the term "musculoskeletal care" from the title, the abstract, and throughout the body of the manuscript. The review is now clearly and consistently framed around orthopaedics alone.

Comment 2: *Introduction: Why a scoping review not a systematic review? Important to justify this and how the two methods differ.*
Response: We have added a paragraph to the Introduction justifying our choice of methodology. We clarified that a scoping review was chosen because our objective is to broadly map a diverse and rapidly expanding body of literature, identifying the types of digital endpoints currently in use across the entire field of orthopaedics. This differs from a systematic review, which typically aims to answer a narrow, specific clinical question regarding a single intervention's efficacy.

Comment 3: *Review question: States only 'What digital endpoints have been evaluated for their psychometric properties in orthopaedic care?' – this would suggest the authors are only interested in which endpoints have been evaluated, not what the psychometric properties are, and again no*

mention of musculoskeletal care as well as orthopaedic?
Response: We have revised the review question to reflect that we are evaluating both *which* endpoints are used and *what* their specific measurement properties are. The updated question reads: *"Which digital endpoints are currently used as outcome measures in orthopaedic care, and what is the extent and nature of their psychometric validation (validity, reliability, responsiveness, and interpretability)?"* We have also removed the mention of musculoskeletal care here.

Comment 4: *Page 5 Participants: Line 23 describes eligible participants, but then Line 33 also does – this should all be together.*
Response: We have restructured the Participants section to group all descriptions of eligible participants together in a single, cohesive paragraph for better flow and readability.

Comment 5: *Page 5 Participants: Line 46: 'populations in which findings are not clearly transferable to orthopaedic care' – what do the authors mean by this?*
Response: We have clarified this sentence to explicitly define what is excluded. The text now reads: *"Studies will be excluded if the focus is solely on non-orthopaedic conditions (e.g., rheumatological diseases like rheumatoid arthritis, or neurological conditions like stroke), as the functional movement patterns and clinical trajectories in these populations differ significantly from those of orthopaedic patients."*

Comment 6: *Page 6 Concept: It would be helpful to the reader to expand on the exact psychometric properties of interest.*
Response: We have expanded the Concept section to explicitly list the specific psychometric properties of interest based on the COSMIN framework, specifically noting: reliability (including measurement error), validity (criterion and construct), responsiveness, and interpretability.

Comment 7: *Page 6 Context: The authors state 'The focus is on measures intended for practical outcome assessment beyond highly controlled laboratory settings' – what do you mean by this?*
Response: We have clarified this sentence to explain our focus on deployable, real-world technologies. The revised text reads: *"This means that while wearable sensors used in home environments or standard outpatient clinics will be included, studies relying exclusively on complex, stationary laboratory equipment (such as marker-based 3D optical motion capture systems fixed within a biomechanics lab) will be excluded, as these cannot be easily deployed as routine digital endpoints in standard clinical trials or remote patient monitoring."*

Comment 8: *Appendix 1 Search strategy: There are no musculoskeletal care search terms? The current search is limited to orthopaedic terms – if this has already been run then the manuscript needs to reflect that only orthopaedic conditions are of interest.*
Response: As noted in our response to Comment 1, we completely agree. We have adjusted the manuscript title and text to reflect that only orthopaedic conditions are of interest, bringing the manuscript directly in line with our tailored search strategy.

VERSION 2 - REVIEW

Reviewer 1
Name Nicolson, Philippa
Affiliation University of Oxford, Nuffield Department of Orthopaedics, Rheumatology and Musculoskeletal Sciences

Date **21-Sep-2026**

COI

Thanks to the authors for their comprehensive revision of the manuscript, and response to previous comments.

One minor edit - the authors now use the term 'orthopaedic care' throughout the manuscript, but the title still refers to 'orthopaedic surgery' - revise for consistency.

VERSION 2 - AUTHOR RESPONSE

Reviewer 1

5. Terminology in the title

Comment: The authors now use the term ‘orthopaedic care’ throughout the manuscript, but the title still refers to ‘orthopaedic surgery’ – revise for consistency.

Response: Thank you for identifying this inconsistency. The title has been revised from “Digital Endpoints as Outcome Measures in Orthopaedic Surgery and Their Psychometric Properties: A Scoping Review Protocol” to “Digital Endpoints as Outcome Measures in Orthopaedic Care and Their Psychometric Properties: A Scoping Review Protocol”, ensuring consistency with the terminology used throughout the manuscript.
